# Integrated Analysis of Gene Expression, Protein Synthesis, and Epigenetic Modifications in *Alcanivorax borkumensis* SK2 Under Iron Limitation

**DOI:** 10.1111/1758-2229.70106

**Published:** 2025-06-02

**Authors:** Francesco Smedile, Renata Denaro, Francesca Crisafi, Domenico Giosa, Giuseppe D'Auria, Manuel Ferrer, Riccardo Rosselli, Martin S. Staege, Michail M. Yakimov, Laura Giuliano, Oleg N. Reva

**Affiliations:** ^1^ National Research Council (CNR) Institute of Polar Sciences (ISP) Messina Italy; ^2^ National Research Council (CNR) Water Research Institute (IRSA) Rome Italy; ^3^ Department of Clinical and Experimental Medicine University Hospital of Messina Messina Italy; ^4^ Spanish Consortium for Research on Epidemiology and Public Health (CIBERESP) Madrid Spain; ^5^ Sequencing and Bioinformatics Service Fundació per al Foment de la Investigació Sanitària i Biomédica de la Comunitat Valenciana (FISABIO) Valencia Spain; ^6^ The Spanish National Research Council (CSIC) Institute of Catalysis Madrid Spain; ^7^ Dpto. Fisiología, Genética y Microbiología Universidad de Alicante Sant Vicent del Raspeig Spain; ^8^ Department of Pediatrics I Martin Luther University Halle‐Wittenberg Halle (Saale) Germany; ^9^ Mediterranean Science Commission (CIESM) Monaco Monaco; ^10^ Department of Biochemistry, Genetics and Microbiology, Centre for Bioinformatics and Computational Biology University of Pretoria Pretoria South Africa

**Keywords:** *Alcanivorax borkumensis* SK2, epigenetics, iron metabolism, methylomics, proteomics, transcriptomics

## Abstract

This study aimed to understand the genetic and molecular mechanisms enabling *Alcanivorax borkumensis* SK2, a hydrocarbonoclastic marine bacterium, to thrive under iron‐limited conditions. Using SMRT PacBio whole‐genome sequencing, Illumina total RNA sequencing, and proteomics analysis, we examined the strain's response to iron‐rich and iron‐depleted media. Despite minimal impact on growth, significant changes in gene expression were observed when using n‐tetradecane or acetate under iron limitation. Iron scarcity, depending on the carbon source, affects energy metabolism, membrane transport, lipid metabolism, stress‐adaptive responses, and siderophore synthesis. We identified several methyltransferases (MTases) in the studied genome, including RS14230, which is a part of a fully functional restriction‐modification (RM) system causing bipartite cytosine methylation and DNA cleavage at AgGCcT sites. Another MTase, RS09425, controls bipartite adenine methylation at GaTNNNNNGtGG motifs; however, no restriction activity at these motifs has been detected. Many epigenetically modified nucleotides lacked canonical motifs, possibly due to MTase byproducts. Notably, non‐canonical modifications were statistically associated with transcriptional start sites and gene regulation, suggesting an indirect role in transcription via DNA conformation changes and its accessibility to MTases near actively transcribed genes.

## Graphical abstract

Summarised transcriptomics, methylomics, and proteomics data plotted to circular maps using Circos software. Meanings of circles and histograms on the plots are explained in the figure legend.

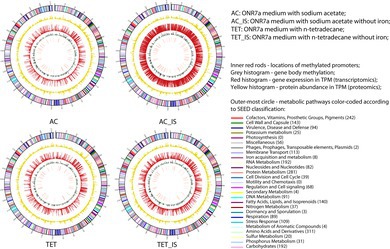

## 1 Introduction

*Alcanivorax borkumensis* SK2 is an obligate marine hydrocarbon‐degrading bacterium (OMHCB) belonging to gamma‐proteobacteria, isolated using n‐alkanes as the sole carbon source, from seawater and sediment samples collected on Borkum Island in the North Sea (Yakimov et al. 1998, 2007). As described previously (Yakimov et al. 2022), the genus *Alcanivorax* comprises 12 recognised and one recently proposed species, *A. indicus* (Song et al. 2018). Like other OHCBs, *A. borkumensis* SK2 plays a crucial role in the degradation of hydrocarbons in marine environments and is among the dominant organisms capable of thriving in acute and chronic oil pollution in the sea (Denaro et al. 2005; Schneiker et al. 2006; Yakimov et al. 2007, 2022). Recently, OHCBs have also been recognised for their significant role as primary colonisers of the plastisphere (Crisafi et al. 2022; Yakimov et al. 2022), contributing to biodegradation processes that can impact petroleum‐derived plastics released into the sea (Cairo et al. 2006; Cao et al. 2022).

*A. borkumensis* SK2 is a cosmopolitan constituent of pelagic bacterioplankton capable of generating substantial blooms in response to the presence of alkane‐hydrocarbons, which are used as carbon sources. This species competes with other microbes for oligonutrients such as iron, which can become scarce during acute or chronic oil spills (Denaro et al. 2014). Nearly all microorganisms rely on iron for their growth. Although Lactobacilli can grow and survive under iron‐limitation conditions (Archibald 1983; Duhutrel et al. 2010), recent studies have shown that increased iron concentration enhances their growth (Huynh et al. 2022).

Iron is a vital component in various metabolic pathways, acting as a cofactor for numerous enzymes and regulatory proteins involved in processes such as the tricarboxylic acid cycle (TCA), electron transport, amino acid biosynthesis, and DNA synthesis (Cairo et al. 2006; Konhauser et al. 2011; Behnke et al. 2023).

Iron limitation in marine pelagic environments is a significant constraint for microbial life. In the aerobic and slightly alkaline pH conditions characteristic of marine environments, iron primarily exists as insoluble ferric hydroxide complexes, leading to its low bioavailability within the water column (Behnke et al. 2023). The typical iron concentration required for optimal bacterial growth ranges between 0.3 and 1.8 μM (Kim et al. 2009). However, concentrations of dissolved iron in offshore waters of the Pacific, Atlantic, and Southern Oceans average only about 0.1 and 0.4 nM at the surface and up to 200 m, and 0.6 and 0.7 nM at meso and bathypelagic depths (Kuma et al. 2003; Kaupp et al. 2011).

The iron requirement of *A. borkumensis* SK2, as for other hydrocarbon‐degrading bacteria, is closely associated with the activity of iron‐dependent and heme‐dependent enzymes crucial for hydrocarbon degradation, including alkane monooxygenase AlkB2 and cytochrome P450 (CYP). AlkB2 is a non‐heme Fe–Fe alkane monooxygenase (Ji et al. 2019), while cytochrome P450 belongs to the alkane hydroxylase family, specifically CYP153 (van Beilen et al. 2006). Moreover, such heme‐dependent enzymes as catalases and peroxidases play a key role in mitigating oxidative stress, which may occur during hydrocarbons biodegradation processes (Alaidaroos 2023). Iron is also essential in Fe‐S cluster enzymes, such as Rieske‐type oxygenases involved in the hydroxylation of aromatic and aliphatic hydrocarbons, or ferredoxin, which facilitates electron transfer during the terminal hydroxylation of alkanes (Sabirova et al. 2011).

Due to the limited availability of iron in marine environments, marine bacteria employ two primary strategies for adaptation in iron‐limiting conditions (Koedooder et al. 2018). Firstly, they can enhance the production of Fe‐uptake molecules such as siderophores and increase the diversity of Fe‐transporter proteins (Sigel and Payne 1982; Tortell et al. 1999; Gledhill and Buck 2012; Wiens et al. 2014; Sasnow et al. 2016). Secondly, bacteria can reduce their dependence on Fe‐containing enzymes by minimising their Fe‐quota. In the genome of *Alcanivorax*, two genes involved in the synthesis of siderophore‐type secondary metabolites were identified: RS10760 and RS09205. RS10760 is involved in the production of amphiphilic siderophores known as amphibactin (Kem et al. 2014), while RS09205 encodes a pseudomonine‐like siderophore (Denaro et al. 2014).

Regulation of metabolic pathways in *Alcanivorax* under iron limitation is not well understood. Even less is known about the possible involvement of epigenetic mechanisms in gene regulation of marine bacteria. This type of soft inheritable genetic regulation involves the arrangement of patterns of gene expression in response to environmental stimuli without altering the genetic material itself. This process is reversible and can bring significant benefits to subsequent generations (Casadesús and Low 2006; Felsenfeld 2014; Chen et al. 2014; Boulias and Greer 2022; Graf et al. 2023). Epigenetics encompasses modifications to genomic DNA at the nucleotide level, such as the addition of small molecules like methyl groups to nucleotides, as well as conformational changes in the chromosome as a macromolecule, making some chromosomal regions more accessible for transcription than others. These modifications can potentially alter protein‐DNA binding affinity, thereby changing the biochemical landscape of DNA, which directly affects the phenotype (Browning et al. 2010; Krogh et al. 2018; Dame et al. 2020).

Epigenetic regulation has been described primarily in eukaryotes, where it is directly involved in cell differentiation or disease linked to altered histone modifications or DNA methylation (Sánchez‐Romero and Casadesús 2020). In recent years, epigenetics has received increasing attention in the field of microbiology. This is because it is directly involved in several biological processes, including genome structure and stability, stress response, cell growth, metabolism, biofilm formation, virulence, and antibiotic resistance (Chen et al. 2014; Adhikari and Curtis 2016; Nye et al. 2019; Sánchez‐Romero and Casadesús 2020; Reva et al. 2020, 2024). Evidence for the existence of epigenetic regulation in prokaryotes is increasing, and this phenomenon appears to be predominantly associated with DNA methylation. DNA methylation is performed by methyltransferases (MTases), which catalyse three types of DNA methylation modifications: N6‐methyl‐adenine (m6A), C5‐methyl‐cytosine (m5C) and N4‐methyl‐cytosine (m4C). While all three types are common in archaea and bacteria, m4C modification is not found in eukaryotes, and m6A is extremely rare. MTases are present in nearly all bacterial species. The majority of bacterial genomes analysed to date exhibit a species or strain‐specific DNA methylation pattern (Blow et al. 2016; Korotetskiy et al. 2023). MTases compose restriction–modification (RM) systems, which consist of a restriction endonuclease (REase) and a MTase with the same DNA binding specificity. The REase degrades DNA lacking methylation at target sites, such as those from bacteriophages and other exogenous sources, while the cognate MTase methylates potential REase target sites in the host genome, protecting them from cleavage. RM systems are categorised into four main types based on the number of subunits, their DNA recognition specificity, and the distance between the DNA binding and cleavage regions (Blow et al. 2016).

In this work, we suggest *A. borkumensis* SK2 as a suitable model to study gene regulation under iron limitation on media with different carbon sources and possible involvement of epigenetic modifications in this regulation. These tasks were achieved by integrating transcriptomics, methylomics, and proteomics data obtained at different growth conditions.

## 2 Materials and Methods

### 2.1 Strain and Culture Conditions

The bacterial strain used in this study was *A. borkumensis* SK2 (ATCC 700651, Yakimov et al. 1998). The strain was routinely grown on ONR7a (Dyksterhouse et al. 1995) agar plates or ONR7a liquid medium supplemented with 0.5% (w/v) of sodium acetate or 0.1% (w/v) of n‐tetradecane.

Iron‐restricted bacterial growth was performed in ONR7a (–Fe) without the addition of FeCl_2_ x 4H_2_O that is normally used as an iron source for this medium. All glassware used was previously treated with 1 M HCl to remove iron traces and then rinsed with bi‐distilled water pH 7.0. To obtain cultures under iron depletion conditions, cells were grown in complete ONR7a medium for 8 days, then harvested and washed four times with ONR7a (without Fe) medium, pelleted by centrifugation (5000 × g) and finally inoculated in ONR7a (–Fe) medium. To test the effect of iron depletion on *A. borkumensis* , growth parameters were estimated by fitting the data to the Gompertz model. Specifically, the maximum optical density at 600 nm (OD_600_), the specific growth rate (*μ*), and the duration of the lag phase were estimated following the method described by Zwietering et al. (1990). All experiments were carried out in triplicate using biological replicates and technical replicates.

### 2.2 Restriction Activity of *A. borkumensis* SK2

A single colony of *A. borkumensis* SK2 grown on ONR7a agar supplemented with n‐tetradecane was transferred into 250 μl of lysis solution (100 mM Tris–HCl pH 8.0, 50 mM NaCl 1 M, 5 mM EDTA, 0.1% Triton X‐100, lysozyme 100 mg L^−1^). The mixture was then incubated at room temperature with stirring for 15 min. The resulting cell lysates were clarified by centrifugation at 5000 × g for 10 min at 4°C. Subsequently, 4.7 μl of cell lysate and 0.2 μl of Lambda DNA were transferred in 20 μl of restriction buffer (20 mM Tris–HCl pH 7.5, 50 mM NaCl 1 M, 10 mM MgCl_2_, 1 mM DT). After incubation at 37°C for 2 h, the restriction products were checked by electrophoresis on a 1.0% (w/v) agarose gel.

### 2.3 RNA Extraction and Sequencing

RNA was extracted from 2 mL of *A. borkumensis* SK2 pure cultures at the mid‐log phase of growth, estimated from growth curves created under the described conditions, using the MasterPure Complete DNA and RNA Purification Kit (Epicentre). The cells were harvested by centrifugation at 5000 × g at 4°C. Extraction was carried out according to the manufacturer's instructions. RNA was stored in isopropanol at −20°C before precipitation. RNA was resuspended in 50 μL of RNase‐free water. Extracted RNA was treated with a TURBO DNA‐free kit (Ambion) to eliminate any residual DNA from the final elution. RNA samples quality and concentration were determined using a Qubit 3.0 fluorometer (Thermo Fisher Scientific, Italy). The integrity and quality of RNA samples were assessed using the Agilent 2100 Bioanalyzer from Agilent Technologies. Following rRNA depletion and DNAse treatment, sequencing was conducted by FISABIO in Valencia, Spain, using the Illumina NextSeq Mid Output platform with 2 × 100 bp short insert paired‐end libraries (NextSeq Reagent Kit v2.5). FISABIO was responsible for both quality assessment and sequence joining, wherein forward R1 and reverse R2 reads were combined.

### 2.4 Differential Gene Expression Analysis

Transcriptional analysis was conducted using Bioconductor 3.17 (http://www.bioconductor.org/) withing R environment v3.4.4. RNA sequences were aligned against the reference genomes indexed by Rsubreads and then sorted with Samtools‐1.18. Reads overlapping predicted coding sequences (CDS) were counted using the *featureCounts* function in Rsubread. DESeq2 and GenomicFeatures normalised counts by total reads and CDS lengths to generate gene expression statistics (baseMeans), expression fold changes, estimated *p*‐values, and *p*‐values adjusted using the Benjamini‐Hochberg procedure. Genes exhibiting a twofold or greater expression difference and a *p*‐value ≤ 0.05 were considered significantly regulated, as these thresholds corresponded to significant Benjamini‐Hochberg adjusted *p*‐values at an alpha level of 0.05.

Gene expression levels in different experiments were compared using reads per kilobase per million mapped reads (RPKM) and transcripts per million (TPM) values, calculated as:

$$\begin{array}{c} \text{RPKM}=\text{number}\_\text{of}\_\text{reads}\_\text{mapped}\_\mathrm{per}\_\text{gene}\times 10^{9}/\\ \;\text{total}\_\text{mapped}\_\text{reads}/\text{gene}\_\text{length} \end{array}$$

For TPM, first normalised read counts (R) were calculated:

R=number_of_reads_mapped_per_gene/gene_length

Then TPM values were calculated for each *i*'s gene:

$$\mathrm{TPM}=10^{6}\times R/\Sigma \left(R_{i}\right)$$

Grouping of gene expression landscapes according to estimated TPM values was performed using Principal Component Analysis (PCA) implemented in PAleontological STatistics (PAST) 4.02 (http://folk.uio.no/ohammer/past/) (Hammer et al. 2013).

For a more effective perceptual comparison of alternative gene regulation under different conditions, the GEMusicA sonification algorithm (Staege 2015, 2021) was used with the first 22 bars form L. van Beethoven's piano piece WoO 59 (‘Für Elise’) as reference melody. GEMusicA decryption scores were calculated essentially as described by Staege (2021) and validated using *t*‐test analysis.

### 2.5 Gene Ontology (GO) Prediction

The Gene ontology (GO) prediction and enrichment analysis of the co‐expressed genes was performed using ShinyGO v0.61 (Ge et al. 2020) with a *p*‐value cutoff set for the false detection rate (FDR) of 0.05.

The prediction and the identification of RM systems and DNA methyltransferases identification (together with the recognition of their cleavage sites) were performed using REBASE (http://rebase.neb.com/rebase/rebase.html) (Roberts et al. 2023).

Summarised transcriptomics, methylomics, and proteomics data were plotted to circular maps using Circos software package v0.69‐9 (Krzywinski et al. 2009).

### 2.6 Protein Extraction and Analysis

The abundance of proteins involved in siderophore biosynthesis was analysed using proteomics. Cell lysis, protein extraction, and proteolytic digestion were conducted following previously established methods (La Cono et al. 2020).

The peptides obtained were subjected to further analysis using a reversed‐phase liquid chromatography mass‐spectrometry method (RP‐LC‐ESI‐MS/MS) employing an EASY‐nLC 1000 System coupled with the Q‐Exactive ultra‐high‐field mass spectrometer (HF‐MS).

Initially, peptides were loaded onto an Acclaim PepMap 100 Trapping column (Thermo Scientific, 20 mm × 75 μm ID: 3‐μm C18 resin with a 100 Å pore size) using buffer A (mobile phase A: 2% acetonitrile [v/v], 0.1% formic acid [v/v]). Subsequently, separation and elution were achieved using a C18 resin analytical column Easy Spray Column (Thermo Scientific, 500 mm × 75 μm, ID: 2‐μm C18 resin with a 100 Å pore size) equipped with an integrated spray tip.

A gradient of 2%–40% Buffer B (100% acetonitrile, 0.1% formic acid [v/v]) in Buffer A was applied over 120 min at a constant flow rate of 250 nL/min. Data acquisition was carried out using a Q‐Exactive HF‐MS with an ion spray voltage of 1.8 kV and an ion transfer temperature of 270°C. All data were acquired using data‐dependent acquisition (DDA) in positive mode with Xcalibur 4.1 software. For MS2 scans, the top 15 most‐abundant precursors with charges ranging from 2+ to 6+ in MS1 scans were selected for higher energy collisional dissociation (HCD) fragmentation, with a dynamic exclusion of 27 s.

MS1 scans were acquired in the m/z range of 390–1700 Da with a mass resolution of 60,000 and automatic gain control (AGC) target of 3E6 at a maximum Ion Time (ITmax) of 20 ms. The threshold to trigger MS2 scans was 8E3; the normalised collision energy (NCE) was set to 27%. Resolved fragments were scanned at a mass resolution of 15,000 with an AGC target value of 2E5 in an ITmax of 120 ms.

Peptide identification from the raw data was conducted using the MASCOT v2.4 search engine within the Protein Discoverer 2.4 Software from Thermo Scientific. The database search was performed against the *A. borkumensis SK2* genome sequences.

The search parameters included tryptic cleavage with up to two missed cleavage sites allowed. Tolerances of 10 ppm for precursor ions and 0.02 Da for MS/MS fragment ions were applied.

FDR scores were adjusted using a percolator algorithm. Protein identifications were accepted based on FDR scores < 1% and the identification of at least one peptide with high confidence.

### 2.7 SMRT PacBio Genomic DNA Sequencing and Methylomics Analysis

Whole genome sequence of the strain *A. borkumensis* SK2 (ATCC 700651) is available at NCBI under accession number NC 008260 (Schneiker et al. 2006). SMRT PacBio sequencing of genomic DNA was used in this study for epigenetics. Sample preparation and sequencing were conducted at GATC Biotech (Constance, Germany) using the PacBio RSII system (Pacific Biosciences, San Diego). PacBio libraries with an 8–12 kb insert size were prepared and sequenced on 1–2 SMRT (Single Molecule, Real‐Time) cells with a 120‐min movie length and MagBead loading. For each sample, an average coverage of approximately 100× per genome was achieved (with a minimum of > 25× per strand). Epigenetic modifications were identified using programs of the smrtlink_12.0.0.177059 package. PacBio reads from samples grown under various conditions were mapped against the reference genome NC_008260 using the *pbmm2 align—sort* algorithm. Epigenetically modified nucleotide and canonical DNA methylation recognition motifs were identified in the resulting BAM files of aligned reads by the programs ipdSummary and motifMaker of the same package as explained in a previous publication (Reva et al. 2024).

### 2.8 Other Bioinformatic Procedures and Statistical Approaches

Metabolic pathways and reactions controlled by differentially expressed genes were identified using Pathway Tools v26.0 (Karp et al. 2002).

The biased distribution of methylated nucleotides in coding and non‐coding genomic regions, in 120 bp TSC‐upstream regions and in the predicted horizontally acquired genetic islands was validated by calculating *z*‐scores, (*F*_obs_ − *F*_exp_)/SQRT(*F*_exp_ + 1), where *F*_obs_ and *F*_exp_ are observed and expected frequencies of modified bases within respective regions. The statistical significance of *Z*‐deviations was confirmed by estimating *p*‐values using the survival function (SF) of the standard normal distribution at ∣*Z*∣, as implemented in the *stats.norm.sf* function of the scipy library. The distribution of methylated motifs across the chromosome and the results of statistical analysis including genetic island identification were visualised by the program SeqWord Motif Mapper v. 3.2.6 (https://github.com/chrilef/BactEpiGenPro).

Spearman correlation between modified base counts within 8 kbp sliding windows with 2 kbp steps and the GC‐content or GC‐skew of the windows was calculated using the *stats.spearman* function of the scipy library. The statistical significance of the calculated correlation was tested by estimating the confidence interval with an alpha of 0.05, using a bootstrap approach with 1000 replicates. Confidence interval boundaries were calculated using the function *percentile* of the numpy library. The correlation coefficient was considered significant if both the lower and upper confidence interval boundaries were either above or below zero.

Statistically reliable associations between nucleotide modifications and downstream gene regulation were identified using contingency tables, validated with the *stats.chi2_contingency* function in Python 3 (scipy v1.11). This function calculates *p*‐statistics for contingency tables and expected frequencies under the assumption of random distribution. Linkage disequilibrium (LD) was estimated as the difference between observed and expected frequencies in the first cell of the contingency table. Then average LD for multiple overlapping sliding windows was calculated.

The observed differences were statistically validated using the binomial test implemented in *stats.binomtest* (SciPy v1.11). To verify the robustness of predictions showing statistically significant associations between methylation in TSC‐upstream loci and the expression levels of downstream genes, the analysis was repeated multiple times with gradually changing sliding window parameters. The window size was adjusted from 20 to 50 bp, increasing by 5 bp in different runs; the number of gene expression categories varied from 4 to 8, increasing incrementally by one; and modified nucleotides were filtered based on NucMod scores, with cut‐off values increasing from 20 to 50 in increments of 5. For each setting, false discovery rate (FDR) values were estimated using the Benjamini‐Hochberg *p*‐value adjustment algorithm (Benjamini and Hochberg 1995), with the alpha set to 0.05. The adjusted *p*‐values varied across different settings, ranging from 0.0002 to 0.0142, with an average of 0.006. In the downstream analysis, associations between alternative base modifications in sliding window loci and downstream gene expression levels were deemed statistically significant when the estimated *p*‐value was equal to or below 0.006.

All these statistical procedures were combined into a Python script pipeline available from Zenodo database (https://zenodo.org/records/13254283).

## 3 Results

### 3.1 Experimental Design

This study examines gene regulation mechanisms in *A. borkumensis* SK2, enabling it to cope with iron‐limiting conditions on two carbon sources: acetate and n‐tetradecane. Acetate, the simplest carbon molecule *A. borkumensis* SK2 can use (Karmainski et al. 2023). n‐Tetradecane, a common alkane in plants and algae, is also a suitable growth substrate for *A. borkumensis* SK2 (Vidyashankar et al. 2015; Cao et al. 2022; Alotaibi et al. 2024). To examine the impact of iron depletion on growth efficiency, *A. borkumensis* SK2 was cultivated in complete ONR7a and iron‐depleted medium (ONR7a –Fe). Sodium acetate and n‐tetradecane served as substrates in both conditions. Transcriptomic, proteomic, and methylomic analyses were conducted on *A. borkumensis* SK2 grown on both substrates to investigate possible mechanisms of its adaptation to iron limitation conditions through gene expression regulation. The sampled omics datasets are listed in Table 1.

**TABLE 1:** Whole genome DNA, total RNA and proteomics datasets obtained from *A. borkumensis* SK2 culture grown on different sole carbon sources and at different iron limitation conditions.

Carbon source	Iron availability	RNA samples	SMRT PacBio samples	Proteomics' samples
Acetate	Available	Ac_A, Ac_B	FAA1, FAA2	Abundance Ratio (log2): AC_(SI)/(I).y
Acetate	Limited	Ac_IS_A, Ac_IS_B	FAB1, FAB2	
n‐Tetradecane	Available	Tet_A, Tet_B	FTA1, FTA2	Abundance Ratio (log2): TET_(SI)/(I).x
n‐Tetradecane	Limited	Tet_IS_A, Tet_IS_B	FTB1, FTB2	

### 3.2 Comparison of Growth Curves of *A. borkumensis* SK2 Under Optimal and Iron Limited Conditions

**FIGURE 1:**
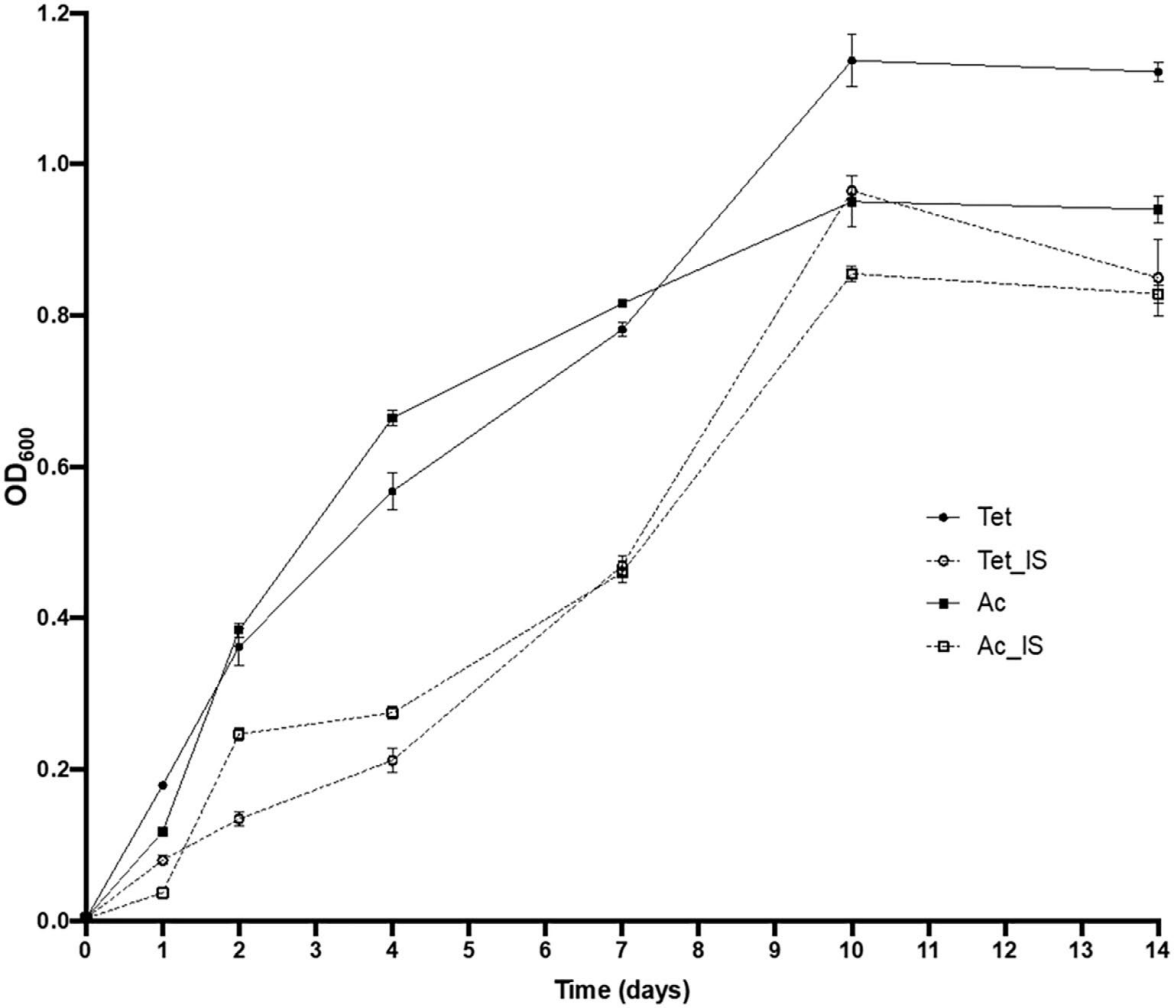
Growth curves of *A. borkumensis* SK2 grown in ONR7a using 0.1% (w/v) of n‐tetradecane (Tet, circle) or 0.5% (w/v) of sodium acetate (Ac, square) as the sole carbon source, and under optimal (solid line) or iron‐limited conditions (IS, dashed line).

The growth curves revealed that *A. borkumensis* SK2 is significantly affected by iron deficiency, regardless of the substrate (Figure 1). This is evident from the prolonged lag phase, which extends to 24 h under iron‐deficient conditions compared to 12 h under optimal growth conditions. With n‐tetradecane as the sole carbon source, we obtained the highest biomass increase, indicating that it is the most favourable substrate, while with acetate we obtained the highest growth rate (*μ*) values due to the rapid metabolism of the substrate, even if the maximum optical density (OD) recorded is lower than that of n‐tetradecane. As shown in Table 2, the lowest *μ* value was observed under acetate with iron deficiency (0.09), followed by n‐tetradecane under iron‐limited conditions (0.11).

**TABLE 2:** Estimation of growth parameters of *A. borkumensis* SK2 under different growth conditions using the Gompertz model.

Growth conditions	Gompertz equation parameters^a^		
	*A*	*μ* (h^−1^)	*λ* (h)
ONR7a + tet 0.1%	1.13 ± 0.03	0.113	12
ONR7a (−Fe) + tet 0.1%	0.96 ± 0.02	0.111	24
ONR7a + Ac 0.5%	0.95 ± 0.03	0.199	12
ONR7a (−Fe) + Ac 0.5%	0.85 ± 0.01	0.09	24

^a^ Parameters of the Gompertz model (± SD): *A*, maximum OD_600_ value reached; *μ*, maximum specific growth rate; and *λ*, lag phase.

### 3.3 Gene Regulation Under Iron‐Limited Conditions

**FIGURE 2:**
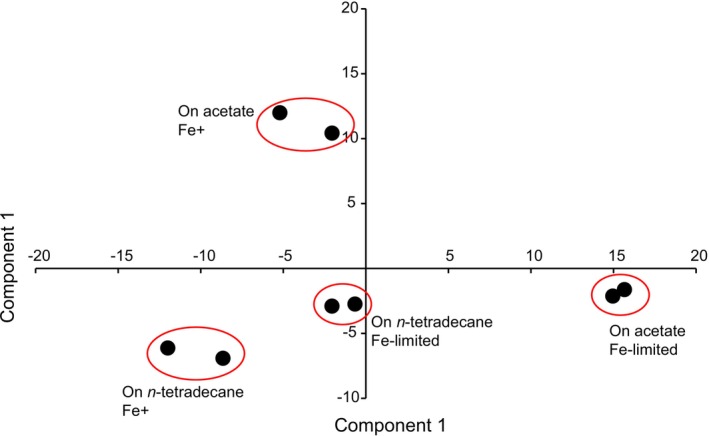
PCA plotting of gene expression patterns of *A. borkumensis* SK2 grown on media with acetate or n‐tetradecane as sole carbon sources under iron‐limited (Fe–) and normal iron concentration (Fe+). Normalised RPKM values of gene expression were used for plotting.

**FIGURE 3:**
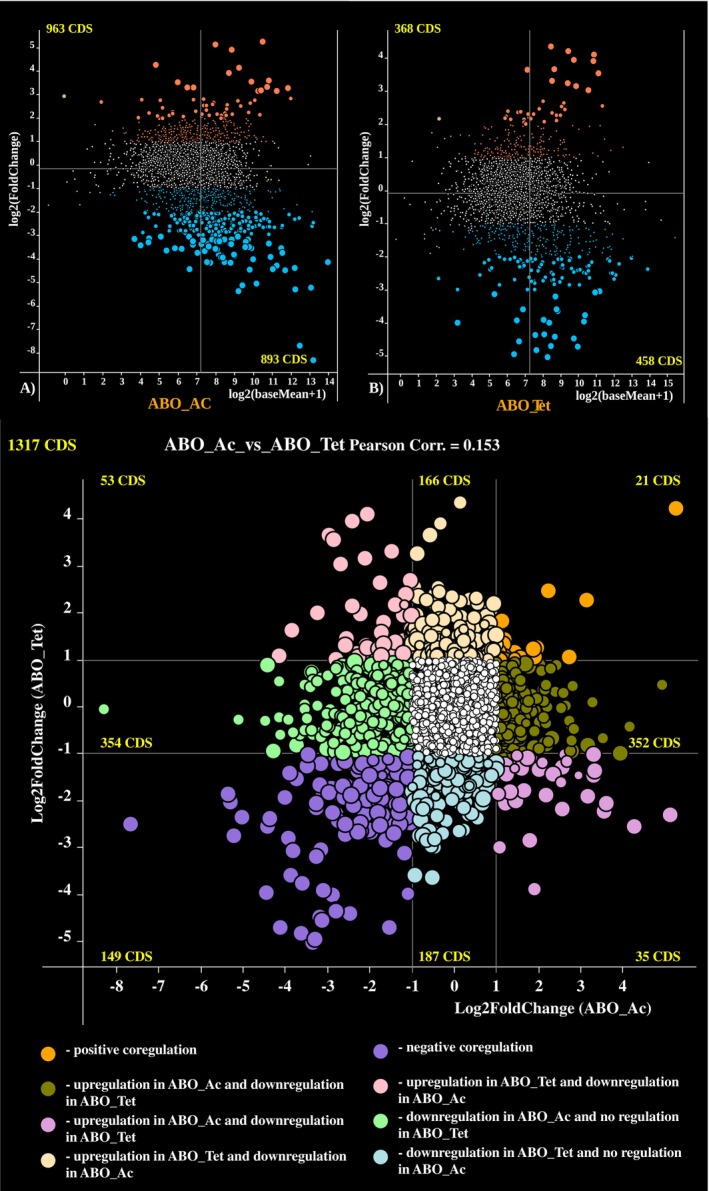
Adaptive gene regulation in *A. borkumensis* SK2 (A) growing in acetate (ABO_Ac) and (B) in n‐tetradecane (ABO_Tet) under optimal and iron limited conditions. Each coloured dot on the plot depicts one gene. Genes were plotted along *X*‐axis according to their mean expression levels estimated as Log_2_(baseMean − 1). Values along *Y*‐axis indicate positive or negative Log_2_(FoldChange) parameters. The genes showing statistically significant up‐regulation under iron limitation (*p* ≤ 0.01 for large and 0.01 < *p* ≤ 0.05 for medium‐sized colour dots) are depicted by orange colour, whereas the negatively regulated genes as shown as blue dots. Regulation of genes depicted by small coloured and white dots was statistically insignificant. (C) Gene co‐regulation analysis was performed by comparison of FoldChange values obtained for the same genes under iron‐limited compared to the normal iron concentration conditions when cultures were grown on acetate and n‐tetradecane as sole carbon sources. Colour code is explained in the figure legend. Numbers of CDS falling to different sectors of the plots are shown.

The transcriptome profile of *A. borkumensis* SK2, growing with two different substrates under optimal and iron‐depleted concentrations, was investigated. Principal component analysis (PCA) plotting of gene expression patterns (Figure 2) showed that the aforementioned ability of *A. borkumensis* SK2 to cope with iron limitation on both carbon sources is achieved through carbon source‐dependent adaptive gene regulation. M‐plots of gene regulation under the two conditions, with the numbers of significantly up‐ and down‐regulated genes, are shown in Figure 3A,B.

Genes that are co‐regulated and counter‐regulated under iron limitation on acetate and n‐tetradecane are shown in Figure 3C and listed in Supporting Information Table S1.

For instance, flavohemoprotein‐encoding gene RS10890 was significantly up‐regulated under iron limitation in both growth conditions. This protein likely plays a significant role in responding to iron limitation, oxidative stress (Remes et al. 2014), iron–sulphur (Fe–S) cluster maintenance (Eijkelkamp et al. 2011), and regulation of intracellular iron homeostasis (Koedooder et al. 2018). Conversely, the pilin protein RS03175, the alginate biosynthesis gene *algS*, and the sigma‐factor *algU*, which are involved in the regulation of biofilm formation and hydrocarbon degradation (Karmainski et al. 2023), were significantly down‐regulated, especially when the culture was grown on acetate. However, under iron‐limitation conditions and using tetradecane as a substrate, we observed an upregulation of functional genes encoding for hydrocarbons biodegradation enzymes.

The major discovery was that iron limitation in the media triggered different responses by *A. borkumensis* strain in terms of gene regulation, depending on the carbon source used in the media (Figures 2 and 3C). The analysis performed by the GEMusicA algorithm (Staege 2021) also suggested a higher divergence of gene expression patterns under iron limitation compared to normal conditions when the strain was grown on acetate rather than on n‐tetradecane: lower decryption scores were found for bacteria grown on acetate (Supporting Information Figure S1) and GEMusicA‐generated melodies (Supporting Information [Link], [Link], [Link], [Link]) showed a stronger distortion of the reference melody (van Beethoven's piano piece WoO 59 ‘Für Elise’) for bacteria grown on acetate under iron limitation.

### 3.4 Gene Ontology Enrichment Analysis of Regulated Genes

**FIGURE 4:**
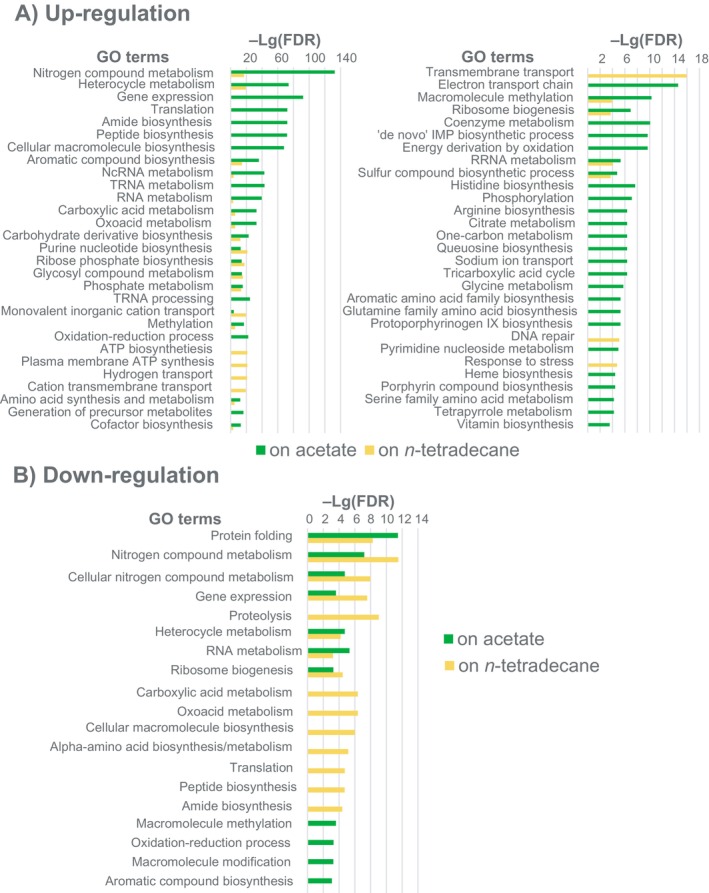
Gene ontology enrichment –*Lg(FRD)* values calculated for (A) up‐regulated, and (B) down‐regulated genes of *A. borkumensis* SK2 under iron limitation on acetate and n‐tetradecane.

Gene ontology enrichment analysis was performed for genes of *A. borkumensis* SK2, regulated under iron limitation conditions on media with acetate and n‐tetradecane as sole carbon sources (Figure 4). Many genes belonging to the same GO categories were regulated oppositely, which may indicate an adjustment of metabolic pathways to stress conditions. The predicted involvement of regulated genes in various metabolic pathways, as predicted by the program Pathway Tools, is shown in Supporting Information Table S1.

### 3.5 Regulation of Genes and Proteins Involved in Siderophore Biosynthesis

There was special interest in the differential regulation of genes encoding siderophores under iron limitation. Two sets of genes involved in siderophore biosynthesis were identified in the *A. borkumensis* SK2 genome: the amphibactin, an amphiphilic siderophore described by Kem et al. (2014), and a pseudomonine‐like siderophore‐producing genes described by Denaro et al. (2014). We found that in the studied conditions, only the operon RS10730–RS10765 encoding synthesis of the amphiphilic siderophore was expressed in *A. borkumensis* SK2. Under iron limitation, these genes were significantly up‐regulated on acetate but showed no differential regulation on n‐tetradecane. Surprisingly, the proteomic analysis showed a significant increase of siderophore biosynthesis proteins under iron limitation in both media (Table 3).

**TABLE 3:** Regulation of genes and proteins involved in siderophore biosynthesis in *A. borkumensis* SK2 grown in Fe‐limited ONR7a medium with acetate or n‐tetradecane, compared to normal growth conditions.

Genes^a^	Gene expression regulation				Protein abundance regulation			
	On acetate		On n‐tetradecane		On acetate		On n‐tetradecane	
	Fold change	*p*	Fold change	*p*	Fold change	*p*	Fold change	*p*
RS10730	−1.16	0.00	−0.45	0.26	Not detected			
RS10735	2.12	0.00	−0.21	0.71	Not detected			
RS10740	4.93	0.00	0.46	0.24	4.02	0.00	2.98	0.00
RS10745	3.93	0.00	−0.99	0.01	Not detected			
RS10750	3.33	0.00	−1.35	0.00	Not detected			
RS10755	2.30	0.00	−0.68	0.04	2.64	0.00	3.42	0.00
RS10760	2.16	0.00	−0.80	0/02	2.37	0.01	3.27	0.00
RS10765	4.26	0.00	−2.54	0.00	Not detected			

*Note:* Fold change of 2 or higher, supported by *p*‐values of 0.05 or less, was considered statistically significant.

^a^ Detailed gene annotation is provided in Supporting Information Table S1.

### 3.6 Restriction‐Modification Systems of *A. borkumensis* SK2

Our assumption that the genome methylation may be involved in gene regulation and stress response necessitated an analysis of the RM systems present in the genome of *A. borkumensis* SK2. Several genes involved in RM systems have been identified using the REBASE database (Roberts et al. 2023). One belongs to the Type I RM system, and two are described as Type II RM (Table 4 and Supporting Information Figure S2).

**TABLE 4:** Genes involved in the RM systems identified in the *A. borkumensis* SK2 genome.

Locus tag	Gene	Most similar	Specificity	Name
Type I				
RS09415		581 aa hypothetical protein		
RS09420	S	Specificity subunit S (EC 3.1.21.3)	GATNNNNNGTGG	S.AboORF1830P
RS09425	M	Type I restriction‐modification system, DNA‐methyltransferase subunit M (EC 2.1.1.72) N6‐adenine DNA methyltransferase	GATNNNNNGTGG	M.AboORF1830P
RS09430		DNA recombination protein RmuC		
RS09435	R	Type I restriction‐modification system, restriction subunit R (EC 3.1.21.3)		AboORF1830P
RS09440		Lipoprotein, putative		
Type II				
RS08590		Hypothetical protein		
RS14230	M	DNA‐cytosine methyltransferase (EC 2.1.1.37)	GGCC	M.AboORF1659P
RS08595		Superfamily I DNA and RNA helicases		
RS10685		Penicillin‐binding protein, putative‐Multimodular transpeptidase‐transglycosylase (EC 2.4.1.129)		
RS10690	M	Putative DNA methylase	AGGCCT	M.AboORF2079P
RS10695	R	Hypothetical protein	AGGCCT	AboORF2079P
RS10700		Transposase subunit B, putative		

The first RM system comprises at least three subunits: a specificity subunit S (RS09420), a class I SAM‐dependent DNA MTase (RS09425), and a REase subunit R (RS09435). Other genes found in different parts of the chromosomes are a DNA‐cytosine MTase (RS14230), a putative DNA MTase (RS10690), and another putative REase (RS10695).

**FIGURE 5:**
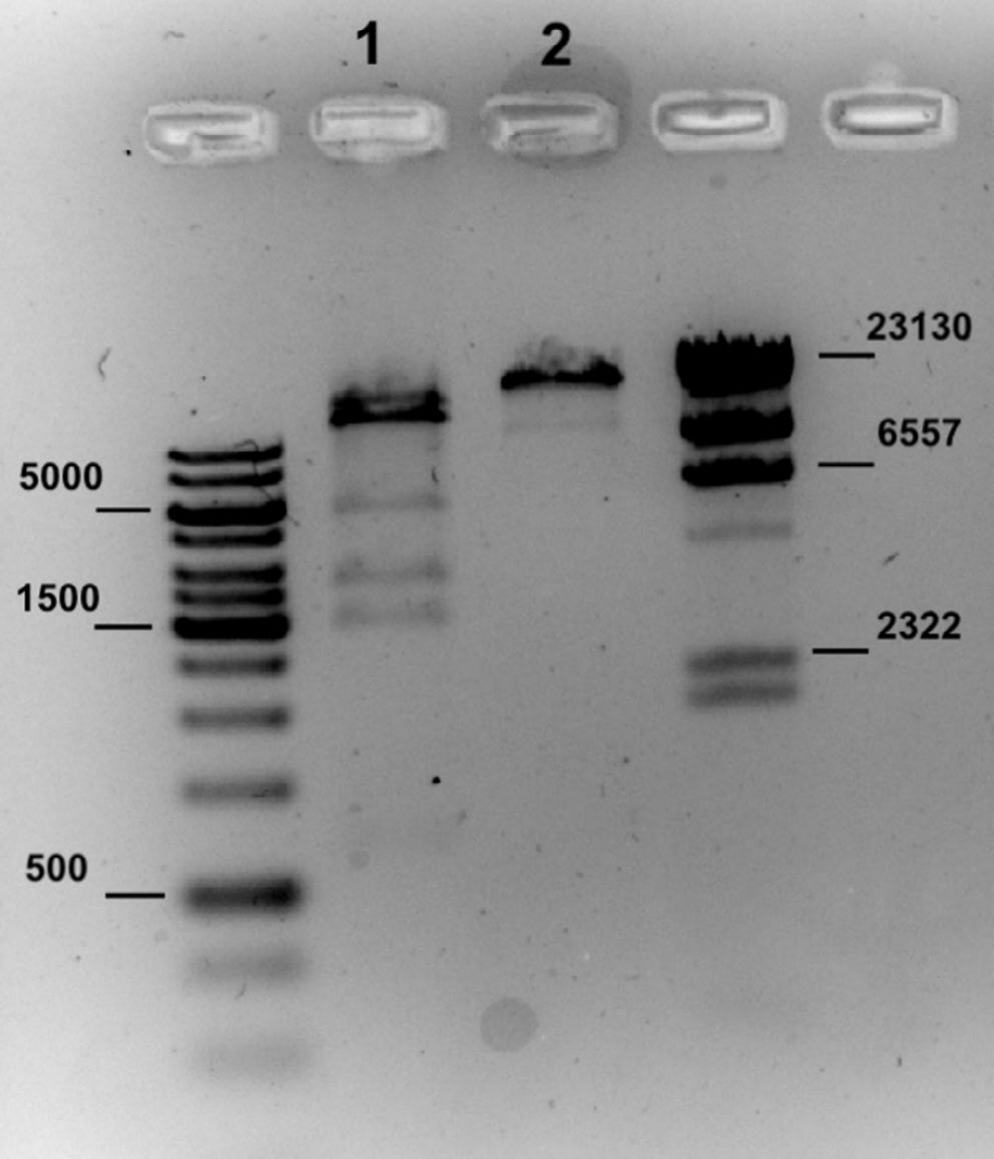
Lambda DNA digested with crude *A. borkumensis* SK2 extract (line 1) and the negative control (2) with the strips of the ladder proteins on both sides.

A total of nine different genomes of *Alcanivorax* species are listed in the REBASE database. Although all of them contain several RM systems, none are similar to the RM systems discovered in the *A. borkumensis* SK2 genome. This suggests the possibility of horizontal acquisition of the RM genes, as previously reported (Jeltsch and Pingoud 1996; Oliveira et al. 2014; Vale et al. 2022; Korotetskiy et al. 2023). The presence of a mobile element protein (RS10700) immediately following the putative MTase (RS10690) and the REase (RS10695) in *A. borkumensis* SK2 supports the hypothesis of horizontal gene transfer. The activity of at least one RM system in *A. borkumensis* SK2 was experimentally confirmed in this study by applying the crude *A. borkumensis* SK2 culture extract to Lambda DNA (Figure 5).

The restriction pattern produced by the *A. borkumensis* SK2 crude extract revealed DNA cleavage exclusively at AGG|CCT sites. This finding demonstrated that only one RM system of *A. borkumensis* was fully functional, suggesting RS10695 to be the active REase according to the REBASE predictions (see Table 4).

It is possible that the MTases of the other RM systems remain active as singular enzymes performing DNA methylation at recognition motifs, while their cognate REases remain inactive despite the observed expression of these genes. Two identified MTases, RS14230 and RS10690, were solitary enzymes with no cognate REases. RS10690 was followed by a gene identified by REBASE as a possible subunit with endonuclease activities. However, this gene does not show any significant sequence similarity with other REases when searched by BLASTP or BLASTX against the NCBI *nr* database. Iron limitation caused twofold down‐regulation of the orphan MTases, RS14230 and RS10690, on acetate, but not on n‐tetradecane.

### 3.7 Patterns of Canonical Methylation

Natural methylation of nucleotide bases in chromosomal DNA was predicted using the program ipdSummary. The program analyses SMRT DNA reads aligned against the reference genome and identifies base locations characterised by unusual base call kinetics in multiple overlapping reads. For detection of epigenetically modified bases, the program utilises the Inter‐Pulse Duration (IPD) and Pulse Width (PW) parameters assigned to each nucleotide in PacBio DNA reads. The program estimates PHRED‐like scores, hereinafter termed NucMod scores, to assess the statistical significance of methylation predictions. Base quality values, sequence depth at each base location, and the fraction of reads exhibiting indicative base call kinetics are all used for statistical validation. Score values above 20 correspond to *p*‐values < 0.01. In this study, all bases with score values above 20 were considered methylated.

Methylation at two canonical motifs, A*g*GC*c*T and G*a*TNNNNNG*t*GG, was identified in the *A. borkumensis* SK2 genome. In both cases, the methylation occurred on both DNA strands, even though the second motif is not a palindrome. Henceforth, the methylated nucleotides, cytosine and adenine, and the respective guanine and thymidine residues opposing the methylated nucleotides on the reverse‐complement DNA strand, are depicted in the canonical motifs by lowercase cursive letters. A*g*GCcT methylation, always present on both strands, was abundant and covered almost all 1674 AGGCCT motifs found in the *A. borkumensis* SK2 genome, leaving only 2–10 motifs unmethylated in different experiments. Nucleotides within canonical motifs were predicted to remain unmodified if their scores, calculated by the program ipdSummary, were below 21. This was the case for the majority of genomic nucleotides. No dependencies between the number or distribution of the unmethylated AGGCCT motifs and the growth conditions were found.

Methylation at G*a*TNNNNNG*t*GG was often incomplete, leaving adenine residues unmethylated predominantly on the complement strand of these motifs. For instance, under iron limitation on n‐tetradecane, the number of unmethylated bases was around 90, similar to that on iron‐rich media with both carbon sources. However, the number of partially methylated motifs increased to 107–118 under iron limitation on acetate (Supporting Information Figure S3).

Partial methylation of both canonical motifs, AGGCCT and GATNNNNNGTGG, suggests that the enzymatic activity of the respective MTase was influenced either by the growth conditions, alterations in DNA conformation, or competition for DNA binding with regulatory proteins produced under specific conditions. Possible associations between alternative patterns of epigenetic base modification and gene expression regulation are discussed in the following section of the paper.

**FIGURE 6:**
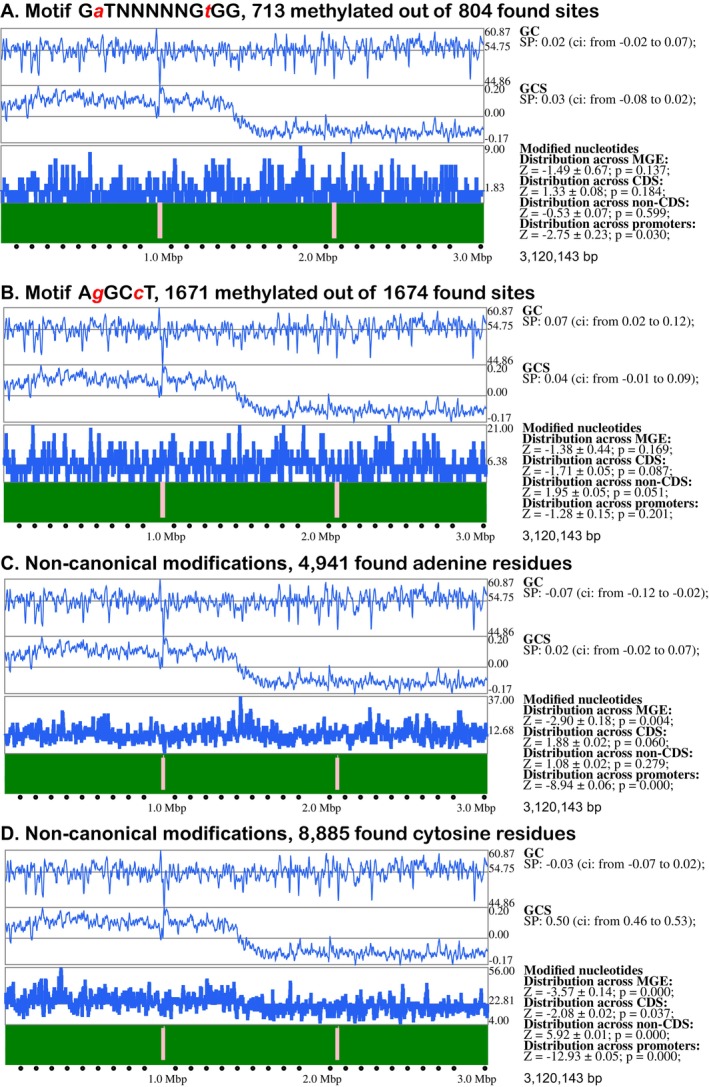
Distribution of methylated canonical motifs and non‐canonical modified bases across various genomic regions predicted by the program SeqWord Motif Mapper for A) GaTNNNNNGtGG motif; B) AgGCcT motif; C) modified adenines; and D) modified cytosines. Each panel represents the distribution of various canonical motifs across the chromosome of *A. borkumensis* SK2. The graphs, from top to bottom, depict GC‐content, GC‐skew, and the number of methylated bases within 8 kbp sliding windows moving along chromosomal sequences with a 2 kbp step. Modified base densities in the lower histograms are depicted by bars extending above and below the average density line. Chromosomal coordinates are shown at the bottom of the panels. Identified inserts of mobile genetic elements (MGEs) are indicated by pink bars. Results of statistical analysis are presented on the right side of the panels, including Spearman correlation (SP) with confidence interval (ci) values between GC‐content and GC‐skew of sliding windows and the number of modified bases within the windows; Z‐scores with standard errors and estimated *p*‐values of biased distribution of modified bases across MGEs and core genomic parts, and between coding, non‐coding, and 120 bp TSC‐upstream regions.

The distribution of methylated motifs A*g*GC*c*T and G*a*TNNNNNG*t*GG was nearly random for A*g*GC*c*T motifs across coding and non‐coding regions. However, methylated G*a*TNNNNNG*t*GG motifs showed statistically significant avoidance in the 120 bp TSC‐upstream regions (*p*‐value 0.03, Figure 6A). Methylation at A*g*GC*c*T motifs was more abundant in non‐coding regions, supported by a *p*‐value of 0.051 (Figure 6B). This observation suggests an evolutionary selection of mutations within these motifs to prevent unnecessary impacts of methylated nucleotides on gene regulation by competing with regulatory proteins binding to DNA in promoter regions.

### 3.8 Distribution of Non‐Canonical Modified Bases

Canonical methylation accounts for approximately 20% of identified epigenetically modified bases predicted by the ipdSummary program. In this study, modified adenine and cytosine residues not associated with canonical motifs were classified as non‐canonical. These modifications may result from inaccurate DNA methylation by MTases outside canonical motifs or the activity of other yet unknown mechanisms.

The distribution of non‐canonical modifications was non‐random and biased, as shown in Figure 6C,D, which represent non‐canonically modified adenine and cytosine residues, respectively. Both modifications were extremely rare in the 120 bp TSC‐upstream regions and in identified mobile genetic element inserts (*p*‐values < 0.004). Non‐canonical cytosine residues were abundant in non‐coding regions and were more frequent on the leading replichore of the chromosome, as indicated by a significant correlation of 0.5 (confidence interval from 0.46 to 0.53) between the frequency of modified cytosine residues in sliding windows and GC skew values calculated for the sliding windows. In contrast, non‐canonical adenine residues exhibited a more random distribution.

**FIGURE 7:**
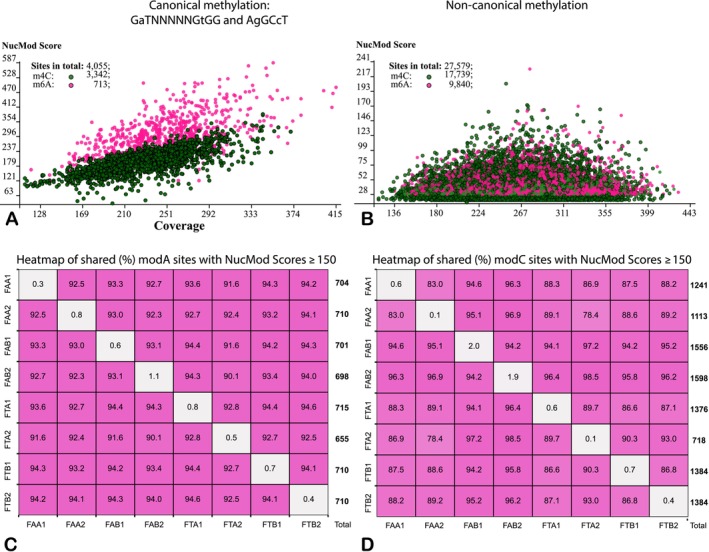
Distribution of canonical and non‐canonical modified adenine and cytosine residues in the *A. borkumensis* SK2 genomes. (A) Canonical methylated nucleotides plotted according to their local coverage values and scores of epigenetic modifications predicted by ipdSummary. (B) Plot of non‐canonical epigenetically modified nucleotides. Individual modified adenine and cytosine residues in both plots are depicted by red and green circles, respectively. (C) Heatmap showing percentages of shared and experiment‐specific (diagonal line) modified adenine residues with NucMod scores ≥ 150. (D) Heatmap of modified cytosine residues with NucMod scores ≥ 150. Total numbers of respective modified nucleotides in each sequenced genome are shown alongside the right vertical edges of the plots.

The biased distribution of non‐canonical modifications across the genome suggests a possible association with differential gene expression. To test this hypothesis, an in‐house Python 3 pipeline was developed for statistical validation. The pipeline is available at https://zenodo.org/records/13254283. This program processes GFF files generated by the ipdSummary program, which contain predicted locations of modified nucleotides and statistics on epigenetic modification calling. NucMod scores, calculated by ipdSummary for multiple overlapping reads, depend on local coverage, as shown in the score‐to‐coverage plots in Figure 7A,B, which were generated for canonical and non‐canonical modified nucleotides, respectively. These plots reveal differences between non‐canonical modifications at adenine and cytosine compared to canonical methylation.

Bases associated with canonical methylation form a cloud‐shaped distribution in the plot, with a linear dependence of NucMod scores on local coverage (Figure 7A). In contrast, non‐canonical modifications follow a Gaussian distribution along the Coverage axis (Figure 7B). Since non‐canonical adenine modifications were predicted solely *in silico*, they are referred to in this paper as epigenetically modified nucleotides rather than methylated nucleotides.

Despite the seemingly random distribution of non‐canonically modified nucleotides, the analysis revealed a strong tendency for the same nucleotides to be repeatedly modified across different experiments. The higher the NucMod score, the greater the likelihood that a given nucleotide would be modified repeatedly. For adenine and cytosine residues with NucMod scores ≥ 150, the repeatability between pairs of experiments was approximately 90%, whereas fewer than 1% of non‐canonical methylated sites were detected only once (Figure 7C,D).

The in‐house pipeline developed for this study employs a sliding window approach to compare changes in epigenetic modification patterns relative to transcriptional start codon (TSC) locations across all genes in the *A. borkumensis* genome, as well as changes in their expression levels (RPKM values) under different conditions. If a genomic region contains more modified bases under one growth condition compared to another, and the downstream genes on the same DNA strand exhibit a significant increase or decrease in expression levels, these observations are recorded in a four‐cell contingency table: [[*a*,*b*],[*c*,*d*]]. Here, *a* represents gene pairs with increased numbers of modified sites correlated with increased gene expression; *b* indicates decreased base modifications correlated with increased expression; *c* denotes increased modified sites correlated with decreased expression; and *d* represents a decrease in both parameters.

To assess the significance of gene expression changes, all genes of *A. borkumensis* were categorised based on their expression levels under a given condition: low, average, or highly expressed. Category boundaries were defined to ensure a similar number of genes in each group. A gene was considered to have a significant change in expression if it shifted from one category to another between conditions.

Biased distributions in contingency tables calculated for sliding windows were analysed using Benjamini‐Hochberg adjusted *p*‐values, allowing a 0.05 FDR, and by linkage disequilibrium (LD) values. A positive LD ≥ 0.5 with a *p*‐value ≤ 0.006 indicates a statistically significant positive association between an increased number of modified bases within genomic regions and increased gene expression. Conversely, a negative LD ≤ −0.5 with a *p*‐value ≤ 0.006 indicates a statistically significant negative association. It was observed that non‐canonical epigenetic modifications of adenine and cytosine residues in several TSC‐upstream and gene‐body regions showed strong associations with positive or negative gene regulation (Supporting Information Figure S4).

**FIGURE 8:**
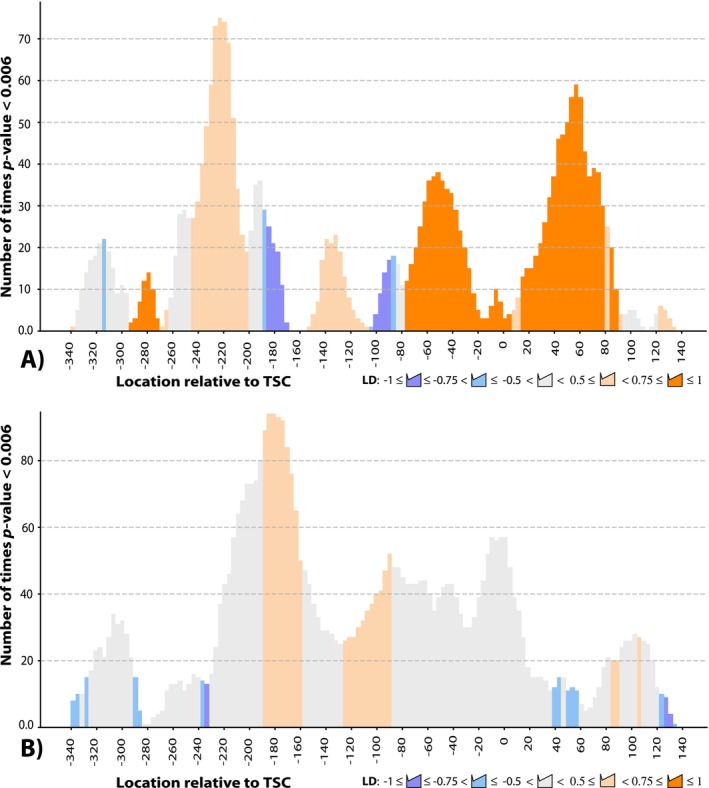
Associations between gene regulation and local non‐canonical epigenetic modifications of (A) adenine and (B) cytosine residues relative to the TSCs of protein‐coding genes. Bars represent the number of times associations between epigenetic modifications and downstream gene regulation reached the Benjamini‐Hochberg adjusted *p*‐values ≤ 0.006 in the respective sliding windows with gradually changed program run settings: Window size was adjusted from 27 to 90 bp, increased by 5 bp in different runs; the number of gene expression categories varied from 3 to 6, increased incrementally by one; and modified nucleotides were filtered based on NucMod scores, with cut‐off values increasing from 100 to 200 in increments of 25. The locations of seven central nucleotides within sliding windows relative to the TSC are shown along the *X*‐axis. Bar colour depicts strong positive (orange) and negative (blue) linkage disequilibrium (LD), indicating associations between changes in the number of modified nucleotides within the sliding windows and expression of downstream genes. Variations in bar colour shade depict different ranges of the average LD‐values, as explained in the figure legend.

The analysis was repeated multiple times with gradually adjusted program settings. The sliding window length varied from 27 to 90 bp, increasing by 5 bp per run. The number of gene expression categories ranged from 3 to 6, increasing incrementally by one. Modified nucleotides were filtered based on NucMod scores, with cut‐off values increasing from 100 to 200 in increments of 25. In total, the program was executed 200 times separately for modified adenine and cytosine residues. Each time a generated contingency table yielded a *p*‐value ≤ 0.006 for any differentially expressed and differentially methylated gene in a pair of experiments (Table 1), the location of seven central nucleotides in the sliding window was recorded. Figure 8 shows the frequency of locations relative to TSC sites within the seven central nucleotides of sliding windows that achieved *p*‐values ≤ 0.006.

Remarkably, strong associations between epigenetic modifications and gene regulation were identified only for nucleotides located at specific loci relative to the TSC of protein‐coding genes. Specifically, an increased frequency of modified adenine residues within 20–80 bp upstream and downstream of the TSC was strongly associated with increased gene expression (LD ≥ 0.75, Figure 8A). Another region with a strong association between the frequency of modified adenine residues and gene expression was located between −240 and −200 bp upstream of the TSC.

These associations were generally positive; however, LD‐values varied from positive to negative probably due to proximity to the region from −200 to −170 bp upstream of the TSC, where negative associations between the frequency of base modification and gene expression were observed.

Epigenetic modifications at cytosine residues showed positive associations with gene expression across a broad region from −230 to +20 bp relative to the TSC locations (Figure 8B). However, the average LD‐values were in the range from 0.5 to 0.75, indicating that in some cases, the detected associations were negative.

Supporting Information Table S2 presents a list of genes significantly regulated under iron limitation on either acetate or n‐tetradecane (see Supporting Information Table S1), which were associated with altered frequencies of modified bases in the TSC‐upstream regions. Modification of cytosine residues showed a stronger association with significant up‐ or down‐regulation of genes. The effect of modified bases on the expression of downstream genes depends on their precise location relative to the TSC sites. This dependence was analysed using contingency tables as explained above. The presence of modified cytosines in [−27.18] region overlapping with the TSC sites was generally associated with down‐regulation: [[*0*,*0*],[*10*,*4*]]. Modifications located from −72 to −315 bp upstream of the TSC showed a positive association between modified cytosines and gene regulation: [[*22*,*8*],[*25*,*49*]], LD’ = 0.51, *p*‐value = 0.0004. The overall contingency table for modified cytosines is: [[*22*,*8*],[*35*,*53*]], LD’ = 0.48, *p*‐value = 0.0026.

## 4 Discussion

*Alcanivorax* genus includes obligate hydrocarbonoclastic bacteria satisfying their metabolic needs by maximising the utilisation of scarcely available carbon sources and outcompeting other microbes for olignutrienst availability. Traces of iron ions in the iron‐limited medium used in this study sufficed for thriving *A. borkumensis* SK2 (Figure 1 and Table 2), but required a significant rearrangement of its metabolism as revealed by the transcriptomic analysis.

Furthermore, to investigate the potential role of epigenetic regulation of gene expression in response to iron limitation, the patterns of canonical and non‐canonical epigenetic modifications of adenine and cytosine residues were analysed and compared to transcriptomic data.

Interestingly, most of the enzymes required for n‐tetradecane utilisation use iron as a cofactor (Ji et al. 2019). Apparently, under conditions of severe iron depletion, the metabolism was rearranged to maximise the salvage of spare resources, which consequently leads to differential regulation of genes involved in iron uptake and a lower growth rate (Supporting Information Table S1).

Gene ontology enrichment analysis led to a surprising discovery that iron limitation has activated several biosynthetic and metabolic processes in *A. borkumensis* SK2, especially when acetate was the sole carbon source (Figures 3A and 4A). Iron limitation is a common condition in the marine environment. It can be assumed that marine bacteria like *A. borkumensis* SK2 have developed proactive metabolic mechanisms to overcome this limitation.

Particularly, genes involved in respiration, glycolysis, gluconeogenesis, TCA cycle, and glyoxylate shunt were up‐regulated under both conditions. This observation corroborates previous findings that iron depletion induces the glyoxylate shunt in heterotrophic bacteria (Koedooder et al. 2018). A substantial number of genes involved in amino acid‐tRNA aminoacylation, folate transformation pathway, and amino acid and nucleotide biosynthesis were significantly up‐regulated. Additionally, numerous metabolic pathways linked to the assimilation of metals through the cell membrane were activated, such as transmembrane transport of metal ions, cellular copper homeostasis, and several pathways associated with iron uptake, including transmembrane transport of iron ions, transmembrane siderophore transport, siderophore uptake activity, and transmembrane heme transport (Tong and Guo 2009).

Specific to growth on acetate was the activation of 2Fe‐2S cluster‐containing cofactor biosynthesis, which plays an essential role in various biological processes such as carbon, nitrogen and sulphur metabolism, respiration, antibiotic biosynthesis, protein translation, replication, DNA repair, gene regulation, and protection from oxidising agents. Furthermore, 2Fe‐2S enzymatic activity centres have been identified as crucial for the assembly of other metal‐containing cofactors (Mendel et al. 2020). Significant activation of peptidoglycan biosynthesis was also specific to this condition. Cultivation of *A. borkumensis* SK2 on n‐tetradecane without iron caused the activation of fatty acid and lipid degradation, β‐oxidation pathways, and the synthesis of cardiolipin. These pathways were down‐regulated on acetate. Other fatty acid and lipid biosynthetic pathways, along with acyl‐carrier protein, were strongly down‐regulated under both conditions. Biosynthetic pathways for biotin and cobalamin were also down‐regulated. Additionally, several metabolic pathways involved in DNA metabolism were found among the down‐regulated genes, such as the regulation of DNA repair, DNA‐mediated transformation, DNA integration, DNA topoisomerase activity, protein‐DNA complex, DNA methylation, and chaperone‐mediated protein folding. Other down‐regulated pathways included the synthesis of adhesion proteins, pilus assembly, ectoine catabolism, response to desiccation, and reactive oxygen species, suggesting an active but suboptimal growth state, indicating some kind of stress affecting *A. borkumensis* SK2.

When acetate is used, *A. borkumensis* SK2 shows a general pattern of overexpression of genes related to different metabolic pathways, particularly those associated with iron uptake and siderophore synthesis. Conversely, when n‐tetradecane is used, only key pathway enzymes are overexpressed. Genes involved in amphiphilic siderophore biosynthesis were differentially regulated in *A. borkumensis* SK2 under iron limitation, depending on the carbon source, despite the observed increase in the abundance of siderophore biosynthesis proteins in both media (Table 3). This may indicate the involvement of regulatory mechanisms acting at the post‐transcriptional or translational level. In line with these findings, Mridha et al. (2024) described a hierarchical and temporally staggered regulation of pyochelin and pyoverdine expression in *Pseudomonas aeruginosa* , reflecting their distinct iron affinities and metabolic costs.

It can be assumed that proteins involved in siderophore synthetase may be stabilised under stress conditions by chaperones or other mechanisms, ensuring their maintenance in bacterial cells without an increase in the expression of the respective genes. Additionally, other authors have reported mechanisms for the recycling of siderophores in *Alcanivorax* (Kube et al. 2013; Denaro et al. 2014). Previous studies have described sophisticated regulatory mechanisms that allow bacteria to sense both iron limitation and the rate of incoming iron‐loaded siderophores to modulate siderophore synthesis (Lamont et al. 2002). This underscores the need to scavenge the scarce iron molecules remaining in the culture medium.

We hypothesized that variations in patterns of epigenetically modified genes may reflect adaptive changes in the expression of genes of *A. borkumensis* SK2 under iron‐limited conditions, or even be involved in gene regulation. The study of patterns of epigenetic modifications in the prokaryotic world is still in its infancy. In particular, there is a lack of understanding of the role of epigenetics in stress response, where, according to a previous publication, epigenetics may play a key role (Mridha and Kümmerli 2022).

Under iron‐limiting conditions in the marine environment, bacteria produce secondary metabolites such as siderophores, and it has been suggested that this process could be under epigenetic control, although its mechanism is still unclear (Giordano et al. 2015; Eirin‐Lopez and Putnam 2019). Other publications have demonstrated epigenetic inheritance in the control of pyoverdine or pyochelin production according to cost‐to‐benefit optimisation (Mridha et al. 2024). Dumas et al. (2013) showed that *Pseudomonas aeruginosa* predominantly invests energy in pyoverdine under stringent iron limitation and switches to increased pyochelin production under moderate iron limitation. Pseudomonine‐like siderophore production could likewise be under epigenetic control with a phase variation switching the related gene on or off. For example, Kube et al. (2013) demonstrated bimodal production of siderophores in *Oleispira antarctica* .

RM systems play a crucial role in prokaryotic defence and are implicated in DNA mismatch repair, gene regulation, and epigenetic rearrangement. Bacterial genomes are dynamic, constantly evolving in response to the interaction between bacteria and their pathogens. This interaction drives the frequent replacement of RM systems with newly acquired genes (Shaw et al. 2023). RM systems, which protect bacteria from foreign DNA, often degrade over time, leaving behind orphan MTases that have lost their associated restriction endonuclease. The orphan MTases have a diminished ability to recognise their original DNA motifs, leading to decreased specificity and function. Moreover, several bacterial MTases, like *dam* MTases in gamma‐Proteobacteria and *ccrM* MTases in alpha‐proteobacteria, which perform bipartite adenine methylation respectively at AGCT and GANTC motifs, seem always to have existed as solitary MTases involved in gene regulation (Marinus and Casadesus 2009). Furthermore, the presence of orphan MTases in bacteria suggests an adaptive mechanism where bacteria maintain some level of DNA methylation to regulate various cellular processes even if the recognition of canonical DNA motifs is reduced (De Ste Croix et al. 2017). This supposition needs a statistical validation that has not been performed till now.

Canonical methylation of the *A. borkumensis* SK2 genome, controlled by an active RM system performing methylation at A*g*GC*c*T motifs and by an orphan MTase performing G*a*TNNNNNG*t*GG methylation, generally was stable under different growth conditions. However, the number of unmethylated adenine residues on the reverse‐complement DNA strand at G*a*TNNNNNG*t*GG motifs increased under iron limitation. The number of differentially methylated canonical nucleotides was insufficient to detect any statistically reliable associations with gene regulation.

In our study, we also analysed the patterns of non‐canonical epigenetic modifications of adenine and cytosine residues. While the nature of these modifications remains disputable, the program ipdSummary predicted modifications at these nucleotides with high statistical reliability. Modifications at the same loci were repeatedly predicted in parallel experiments; however, these patterns were more variable under different growth conditions than canonical methylation. We performed a statistical analysis using the in‐house programs written in Python to verify whether variations in the numbers of epigenetically modified nucleotides within genomic loci upstream and downstream of the TSC of protein‐coding genes are associated with alternative gene expression. For the first time, the associations between differential non‐canonical modifications of nucleotides at distinct locations relative to the TSC and gene regulation were demonstrated in this paper, marking the major discovery of this work. Epigenetic modifications in the genomic regions near the TSC of protein‐coding genes may reflect changes in accessibility of these regions due to conformational rearrangements of the chromosome, but also may stabilise the spatial organisation of the chromosome and/or propagate specific chromosomal folding during replication, enabling epigenetic inheritance across generations (Cuvier and Fierz 2017). Genome architecture landscape and accessibility profiling is a rapidly developing field shedding light on the complexity of transcriptional regulation in prokaryotes, beyond classical transcriptional regulators (Hofmann and Heermann 2015; Minnoye et al. 2020).

## 5 Conclusion

In our research, we found significant associations between alternative methylation near the TSC of genes and variations in gene expression and regulation needed for adaptation of the model microorganism, *A. borkumensis* SK2, to the iron limited condition. However, our statistical analyses could not conclusively determine whether the observed changes in gene transcription are linked exclusively to the epigenetic modification described in this paper or resulted from other regulatory mechanisms or altered chromosomal DNA conformation. A limitation of this study is the unclear chemical nature of the non‐canonical epigenetic modifications, which most likely is a sporadic methylation by available MTases. However, adenine and cytosine methylation at these sites were predicted only *in silico* by the program ipdSummary through computational analysis of SMRT base call kinetics and never has been confirmed experimentally. It is also unclear whether the observed non‐canonical methylation was controlled by RM‐associated MTases or by some unknown mechanisms. Nevertheless, strong statistical associations were demonstrated between gene regulation and non‐canonical epigenetic modifications of adenine and cytosine residues at specific locations relative to the genes' TSC.

Epigenetic modifications in the *A. borkumensis* SK2 genome underline a very complex pattern of gene regulation in this microorganism, allowing it to survive and overcome strict iron limitation in the medium or environment. Our study suggests that analysing the density of epigenetically modified sites near the TSC of protein‐coding genes may provide information on the accessibility states of these loci, regardless of whether the epigenetic modifications are a cause or a reflection of changed genome conformation.

## Author Contributions

**Francesco Smedile:** data curation, software, validation, visualization, project administration, conceptualization, writing – original draft, writing – review and editing, investigation, methodology. **Renata Denaro:** conceptualization, methodology, investigation, writing – review and editing. **Francesca Crisafi:** conceptualization, methodology, investigation, data curation, software, validation, visualization, project administration, writing – review and editing. **Domenico Giosa:** investigation, validation, writing – review and editing, project administration, software. **Giuseppe D'Auria:** investigation, software, validation, project administration, writing – review and editing. **Manuel Ferrer:** methodology, software, data curation, investigation, writing – review and editing, project administration. **Riccardo Rosselli:** methodology, investigation, data curation, writing – review and editing. **Martin S. Staege:** writing – review and editing, investigation, methodology, data curation, visualization, validation. **Michail M. Yakimov:** conceptualization, investigation, funding acquisition, writing – original draft, writing – review and editing, visualization, validation, methodology, formal analysis, project administration, data curation, supervision, resources. **Laura Giuliano:** conceptualization, formal analysis, investigation, writing – review and editing. **Oleg N. Reva:** conceptualization, software, methodology, data curation, investigation, validation, formal analysis, visualization, writing – original draft, writing – review and editing.

## Conflicts of Interest

The authors declare no conflicts of interest.

## Supporting information


**Supporting Information SM1.** Example of a GEMusicA generated melody from gene expression data of *A. borkumensis* SK2 under iron limitation on the medium with acetate (Ac_IS1). Beethoven’s “Für Elise” was used as reference melody.

**Supporting Information SM2.** Example of a GEMusicA generated melody from gene expression data of *A. borkumensis* SK2 under iron limitation on the medium with acetate (Ac_IS2). Beethoven’s “Für Elise” was used as reference melody.

**Supporting Information SM3.** Example of a GEMusicA generated melody from gene expression data of *A. borkumensis* SK2 under iron limitation on the medium n‐tetradecane (T_IS1). Beethoven’s “Für Elise” was used as reference melody.

**Supporting Information SM4.** example of a GEMusicA generated melody from gene expression data of *A. borkumensis* SK2 under iron limitation on the medium n‐tetradecane (T_IS2). Beethoven’s “Für Elise” was used as reference melody.

**Supporting Information Figure S1.** Values of decryption scores obtained with GEMusicA software for *A. borkumensis* SK2 growing in acetate (A) and n‐tetradecane (B), with normal iron‐concentration (replicates 1 and 2) and under iron limitation condiction (replicate IS1 and IS2). (C) Statistical comparison by t‐test of decryption scores obtained from *A. borkumensis* SK2 growing in acetate and n‐tetradecane with normal iron‐concentration and under iron limitation condiction.

**Supporting Information Figure S2.** Location and structure of possible restriction‐modification (RM) operons on the chromosome of *A. borkumensis* SK2. Methyltransferases are shown blue‐dashed; restrictases—red‐dashed; and the S‐protein—green‐dashed.

**Supporting Information Figure S3.** Distribution of G*a*TNNNNNGTGG partially methylated motifs in the *A. borkumensis* SK2 genome under the following conditions: FAA—with iron on acetate; FAB—without iron on acetate; FTA—with iron on n‐tetradecane; FTB—without iron on n‐tetradecane. Genome sequences are shown as circular diagrams going clockwise from the replication origin, depicted by the top vertical line. Fluctuations of GC content and GC skew in 5 kbp sliding windows are shown by blue and black lines. Numbers in the central parts of the circular diagrams show the number of unmethylated nucleotides/expected number of methylated nucleotides (804) within the 402 found GATNNNNNGTGG canonical motifs.

**Supporting Information Figure S4.** Calculated *p*‐values of associations between methylation of (A) adenine and (B) cytosine at locations relative to transcriptional start codons (TSC) and the regulation of the respective downstream genes. Bars on the graph represent contingency table *p*‐values calculated for 36 bp sliding windows with central points relative to TSC locations shown along the X‐axis. Other applied program‐run parameters were: NucMod cutoff ≥ 150 and four gene expression categories. Bar colour depicts generally positive (orange) and negative (blue) linkage disequilibrium (LD) values of associations between changes in the number of modified nucleotides within the window and regulation of downstream genes. Vertical black whiskers illustrate the range of variation in *p*‐values across the three calculations for overalpping sliding window, with a 2 bp positive and negative increments. Locations where the estimated *p*‐value was equal or lower than the Benjamini‐Hochberg adjusted *p*‐value 0.006 are marked by asterisks.

**Supporting Information Table S1.** Co‐reculation and counter‐regulation of genes of *A. borkumensis* SK2 under iron limitation on acetate and n‐tetradecane.

**Supporting Information Table S2.** Associations between epigenetic modifications within TSC‐upstream regions and expression of genes regulated under iron‐limited conditions.

## Data Availability

NCBI BioProject PRJNA1114717 Zenodo https://zenodo.org/records/13254283. doi: https://doi.org/10.5281/zenodo.13254283.
