# Supplementary figures and images for: Integrated Analysis of Gene Expression, Protein Synthesis, and Epigenetic Modifications in Alcanivorax borkumensis SK2 Under Iron Limitation
Source: Environ Microbiol Rep. 2025 Jun 2;17(3):e70106. doi: 10.1111/1758-2229.70106 (PMC12128166; doi:10.1111/1758-2229.70106)

### A) Acetate

|       | A1    | A2    | A_IS1 | A_IS2 |
|-------|-------|-------|-------|-------|
| A1    | 100   | 75.38 | 20.00 | 23.08 |
| A2    | 70.77 | 100   | 0     | 3.08  |
| A_IS1 | 38.46 | 27.69 | 100   | 96.92 |
| A_IS2 | 41.54 | 30.77 | 96.92 | 100   |

### B) n-Tetradecane

|       | T1    | T2    | T_IS1 | T_IS2 |
|-------|-------|-------|-------|-------|
| T1    | 100   | 61.54 | 50.77 | 50.77 |
| T2    | 64.62 | 100   | 66.15 | 63.08 |
| T_IS1 | 58.46 | 73.85 | 100   | 73.85 |
| T_IS2 | 56.92 | 63.08 | 70.77 | 100   |

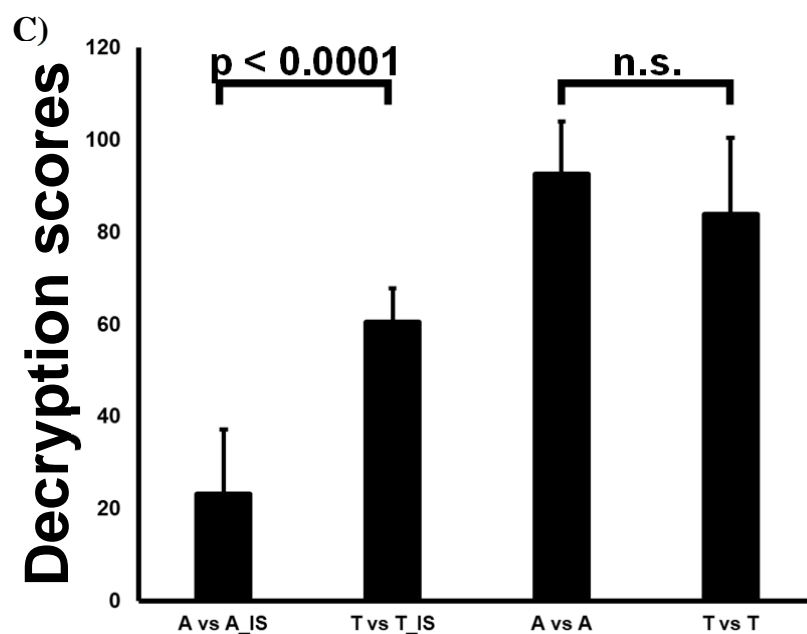

Supplement: Supplementary file 5 — Supporting Information Figure S1. Values of decryption scores obtained with GEMusicA software for A. borkumensis SK2 growing in acetate (A) and n‐tetradecane (B), with normal iron‐concentration (replicates 1 and 2) and under iron limitation condiction (replicate IS1 and IS2). (C) Statistical comparison by t‐test of decryption scores obtained from A. borkumensis SK2 growing in acetate and n‐tetradecane with normal iron‐concentration and under iron limitation condiction. [file EMI4-17-e70106-s008.pdf]

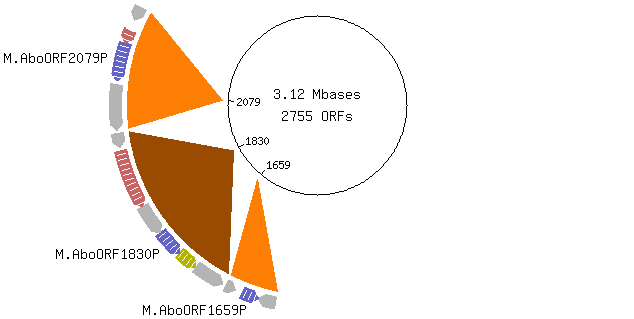

Supplement: Supplementary file 6 — Supporting Information Figure S2. Location and structure of possible restriction‐modification (RM) operons on the chromosome of A. borkumensis SK2. Methyltransferases are shown blue‐dashed; restrictases—red‐dashed; and the S‐protein—green‐dashed. [file EMI4-17-e70106-s005.tif]

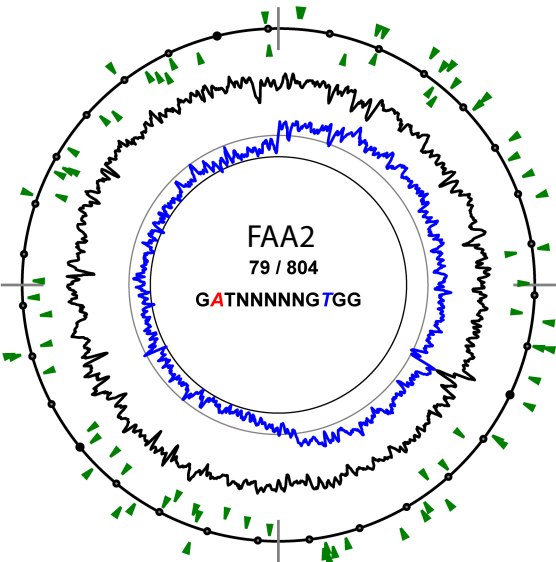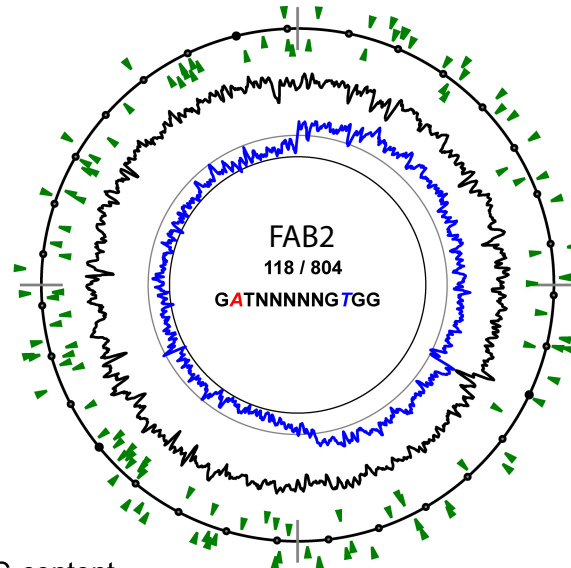

— GC-content  
— GC-skew

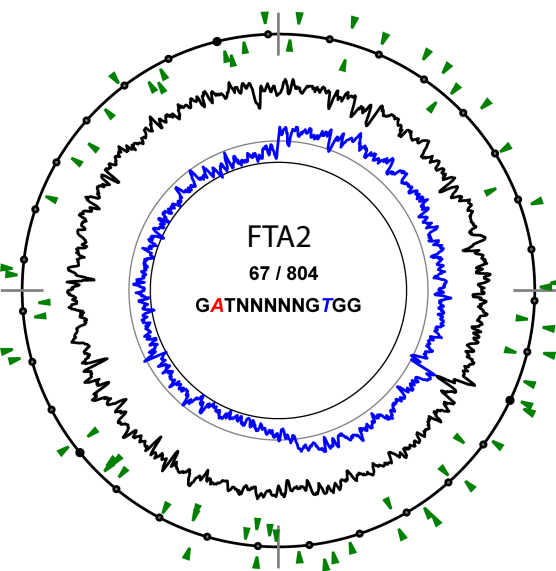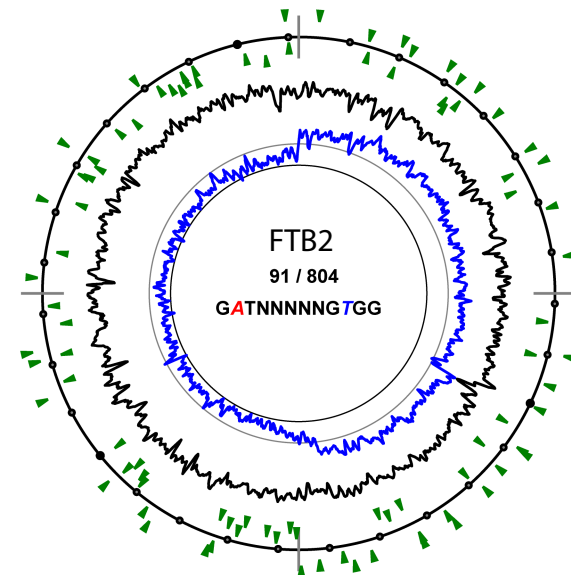

Supplement: Supplementary file 7 — Supporting Information Figure S3. Distribution of GaTNNNNNGTGG partially methylated motifs in the A. borkumensis SK2 genome under the following conditions: FAA—with iron on acetate; FAB—without iron on acetate; FTA—with iron on n‐tetradecane; FTB—without iron on n‐tetradecane. Genome sequences are shown as circular diagrams going clockwise from the replication origin, depicted by the top vertical line. Fluctuations of GC content and GC skew in 5 kbp sliding windows are shown by blue and black lines. Numbers in the central parts of the circular diagrams show the number of unmethylated nucleotides/expected number of methylated nucleotides (804) within the 402 found GATNNNNNGTGG canonical motifs. [file EMI4-17-e70106-s006.pdf]

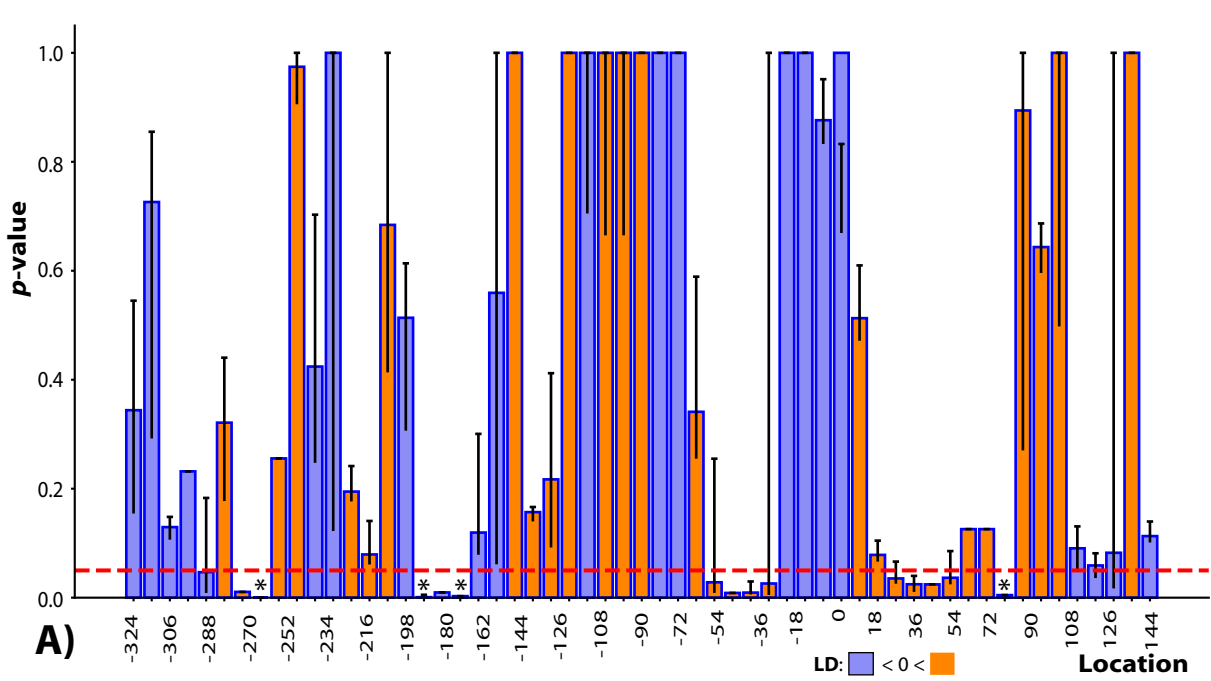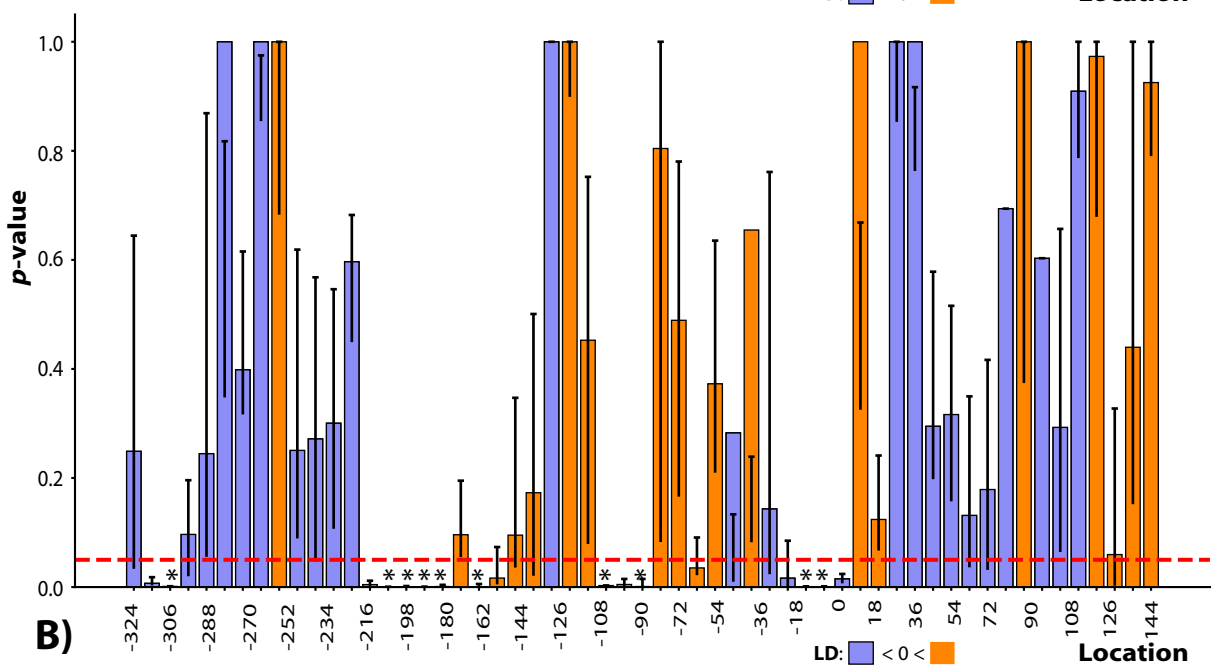

Supplement: Supplementary file 8 — Supporting Information Figure S4. Calculated p‐values of associations between methylation of (A) adenine and (B) cytosine at locations relative to transcriptional start codons (TSC) and the regulation of the respective downstream genes. Bars on the graph represent contingency table p‐values calculated for 36 bp sliding windows with central points relative to TSC locations shown along the X‐axis. Other applied program‐run parameters were: NucMod cutoff ≥ 150 and four gene expression categories. Bar colour depicts generally positive (orange) and negative (blue) linkage disequilibrium (LD) values of associations between changes in the number of modified nucleotides within the window and regulation of downstream genes. Vertical black whiskers illustrate the range of variation in p‐values across the three calculations for overalpping sliding window, with a 2 bp positive and negative increments. Locations where the estimated p‐value was equal or lower than the Benjamini‐Hochberg adjusted p‐value 0.006 are marked by asterisks. [file EMI4-17-e70106-s004.pdf]
